# Comparative Evaluation of the Accuracy of an Electronic Apex Locator Using Two Different Canal Conditions: An In Vitro Study

**DOI:** 10.7759/cureus.108889

**Published:** 2026-05-15

**Authors:** Mohd Zeeshan Ahmad, Divyangana Thakur, Megha Ghosh, Ritika Chaudhary, Anuradha Reddy, Twinkle Gupta

**Affiliations:** 1 Department of Conservative Dentistry and Endodontics, Rama Dental College Hospital and Research Center, Kanpur, IND; 2 Department of Conservative Dentistry and Endodontics, Swami Devi Dyal Hospital and Dental College, Panchkula, IND; 3 Department of Conservative Dentistry and Endodontics, College of Dental Science and Hospital, Indore, IND; 4 Department of Conservative Dentistry and Endodontics, Uttaranchal Dental and Medical Research Institute, Dehradun, IND; 5 Department of Conservative Dentistry and Endodontics, Smiline Dental Hospital, Hyderabad, IND; 6 Department of Conservative Dentistry and Endodontics, Kanti Devi Dental College and Hospital, Mathura, IND

**Keywords:** electronic apex locator, normal saline, root canal irrigant, sodium hypochlorite, working length

## Abstract

Introduction: Accurate determination of the working length is critical for the success of root canal treatment, as it ensures effective cleaning, shaping, and obturation of the root canal system. Electronic apex locators are widely used for this purpose; however, their accuracy may be influenced by intracanal conditions, particularly the presence or absence of irrigation solutions. This study aimed to evaluate and compare the accuracy of a third-generation apex locator under dry, normal saline, and 3% sodium hypochlorite canal conditions.

Materials and methods: This in vitro study was conducted on 45 extracted human single-rooted teeth randomly divided into three groups (n = 15 each) based on canal conditions: dry, normal saline, and 3% sodium hypochlorite. The actual working length (AWL) was determined visually and was used as the gold standard. The electronic working length (EWL) was measured using a Root ZX Mini. The difference between the EWL and AWL was calculated. Data were analyzed using one-way analysis of variance (ANOVA) followed by Tukey’s post-hoc test, and categorical accuracy was assessed using the chi-square test, with significance set at p < 0.05.

Results: The normal saline group demonstrated the lowest mean deviation (0.21 ± 0.14 mm; 95% CI: 0.13-0.29), followed by the sodium hypochlorite group (0.34 ± 0.19 mm; 95% CI: 0.23-0.45), while the dry canal group showed the highest deviation (0.58 ± 0.22 mm; 95% CI: 0.46-0.70). A one-way ANOVA revealed a statistically significant difference between the groups (F = 18.74, p < 0.001). Post hoc analysis revealed significant differences between all groups (p < 0.05). The proportion of accurate measurements (±0.5 mm) was highest in the normal saline group, followed by the sodium hypochlorite and dry canal groups, although the chi-square test was not statistically significant (p = 0.071).

Conclusions: Intracanal conditions significantly influenced the apex locator accuracy. Normal saline provided the most accurate measurements, whereas the dry conditions showed greater variability. Maintaining a moist canal environment may enhance clinical reliability.

## Introduction

Accurate determination of the working length is a critical step in successful endodontic therapy, as it directly influences the effectiveness of canal debridement, disinfection, and obturation [1]. An inadequate working length may lead to incomplete removal of necrotic tissue and microorganisms, whereas over-instrumentation can cause periapical irritation, postoperative pain, and delayed healing [2]. Traditionally, radiographic methods have been employed for working-length determination; however, a previous study has reported increased postoperative pain in patients whose working length was determined using digital radiographs [2]. Consequently, electronic apex locators (EALs) have gained widespread acceptance as reliable adjuncts or alternatives to radiographic methods [3,4].

Third-generation apex locators, such as the Root ZX Mini, operate based on impedance measurements at multiple frequencies and have demonstrated improved accuracy under various clinical conditions [5]. These devices are less influenced by the presence of electrolytes within the root canal system compared with earlier generations [6]. Nevertheless, the accuracy of EALs may still be affected by canal conditions, including the presence of irrigating solutions such as saline, sodium hypochlorite, or chlorhexidine, which alter electrical conductivity within the canal [7,8]. Understanding the influence of different canal environments on apex locator performance is essential for optimizing clinical outcomes.

Several in vitro studies have attempted to simulate clinical conditions to evaluate the precision of EALs, often using embedding media, such as alginate, to mimic the periodontal ligament [9,10]. Despite advancements in technology, the variability in accuracy under different canal conditions necessitates further investigation to validate their clinical reliability. The aim of this study was to comparatively evaluate the accuracy of a third-generation apex locator under different canal conditions (dry, saline, and sodium hypochlorite [NaOCl]) in an in vitro setting. The objective was to determine the electronic working length under each condition, compare it with the actual working length (gold standard), and assess the degree of accuracy within clinically acceptable limits (±0.5 mm and ±1 mm).

## Materials and methods

This in vitro experimental study was conducted in the Department of Conservative Dentistry and Endodontics, Rama Dental College Hospital and Research Centre, Kanpur, India, from June 2025 to November 2025 using freshly extracted human permanent single-rooted teeth obtained from the Department of Oral and Maxillofacial Surgery. Teeth were collected from patients undergoing routine extractions after obtaining informed consent for the use of their extracted teeth for research purposes. Ethical approval was waived by the institutional ethics committee because the study involved extracted teeth with no patient identifiers or direct patient participation.

Teeth scheduled for extraction due to periodontal or orthodontic indications were considered for inclusion. A total of 45 specimens were selected, each exhibiting a fully developed apex, a single root canal, and no signs of root resorption, fractures, calcifications, or prior endodontic intervention. Specimens were excluded if they demonstrated canal curvature exceeding 20^0^, immature apices, or any form of resorptive defects.

The sample size was calculated using G*Power software (version 3.1.9.7; Heinrich Heine University, Düsseldorf, Germany) based on a one-way analysis of variance (ANOVA) with an effect size of 0.40, α = 0.05, and power = 0.80, resulting in 15 samples per group [11]. All adherent soft tissue and calculi were removed using an ultrasonic scaler, and the teeth were stored in 0.1% thymol solution for no longer than one month to prevent dehydration and microbial growth.

The crowns were decoronated at the cementoenamel junction using a diamond disc under continuous water coolant to standardize the root length to approximately 15 mm. Standard access cavities were prepared using endodontic access burs (Dentsply Sirona, Charlotte, NC). Canal patency was established using a size #10 K-file (Mani Inc., Tochigi, Japan), followed by coronal pre-flaring with Gates-Glidden drill sizes #2 and #3 (Dentsply Sirona, Charlotte, NC, USA) to eliminate coronal interference. Canals were irrigated with distilled water and dried using sterile paper points.

The actual working length (AWL) was determined by inserting a size #10 K-file (Mani Inc., Tochigi, Japan) into the canal until the file tip was visible at the apical foramen under 3.5x magnification using dental operating loupes (Univet, Rezzato, Italy). The silicone stopper was adjusted to a reproducible coronal reference point, and the length was measured using a digital Vernier caliper (Mitutoyo Corporation, Kawasaki, Japan) with an accuracy of 0.01 mm. From this measurement, 0.5 mm was subtracted to establish the AWL, which served as the gold standard.

To simulate the periodontal ligament and surrounding supporting structures, each specimen was embedded in freshly mixed alginate impression material (Zelgan 2002, Dentsply Sirona, Konstanz, Germany) contained within plastic molds. A lip clip of the apex locator was inserted into the alginate to complete the electrical circuit. All measurements were performed immediately after embedding to avoid dimensional changes caused by alginate dehydration.

Electronic working length (EWL) was determined using a third-generation EAL (Root ZX Mini [J. Morita Corp., Kyoto, Japan]), in accordance with the manufacturer’s instructions. Each canal was evaluated under three different conditions: dry, saline, and NaOCl. The sequence of the testing conditions was randomized to minimize bias. The canals were thoroughly dried under dry conditions using sterile paper points. Under saline conditions, the canals were irrigated with 2-3 mL of 0.9% normal saline (Baxter Healthcare Corporation, Deerfield, IL, USA), and excess irrigant was removed without complete drying. In the NaOCl condition, the canals were irrigated with 2-3 mL of 3% NaOCl solution (Prime Dental Products Pvt. Ltd., Mumbai, India), and measurements were taken without drying the canal.

A #15 K-file (Mani Inc., Tochigi, Japan) was connected to the apex locator and advanced into the canal until the device indicated the apex. The file was then withdrawn until the display indicated a 0.5 mm short apex, and this length was recorded as the EWL. Each measurement was performed twice, and the mean value was used to minimize variability. All measurements were performed by a single calibrated operator under standardized laboratory conditions at room temperature. The operator was blinded to the AWL during electronic measurements. Intra-examiner reliability was assessed to ensure consistency. The difference between the EWL and AWL was calculated for each specimen. Measurements were categorized as accurate if within ±0.5 mm, acceptable if within ±1 mm, and inaccurate if the deviation exceeded ±1 mm.

All data were recorded in Microsoft Excel (Microsoft Corporation, Redmond, WA, USA) and analyzed using IBM Corp. Released 2020. IBM SPSS Statistics for Windows, Version 26. Armonk, NY: IBM Corp. Continuous variables are expressed as mean ± standard deviation (SD), and 95% confidence intervals (CIs) were calculated. Normality of data distribution was assessed using the Shapiro-Wilk test, and homogeneity of variance was evaluated using Levene’s test. Inter-group comparisons of the mean working length deviation were performed using one-way ANOVA, followed by Tukey’s honest significant difference (HSD) post-hoc test for pairwise comparisons. Categorical data for accuracy classification were analyzed using the chi-squared test. The effect size (partial eta squared) was calculated to determine the magnitude of differences. Intra-examiner reliability was assessed using the intraclass correlation coefficient (ICC). Statistical significance was set at p < 0.05.

## Results

The present study evaluated the accuracy of EWL measurements under three canal conditions: normal saline, 3% NaOCl, and a dry canal. Table 1 presents descriptive statistics of the working length deviation. Among the three groups, the normal saline group demonstrated the lowest mean deviation (0.21 ± 0.14 mm; 95% CI: 0.13-0.29), indicating the highest accuracy. This was followed by the NaOCl group (0.34 ± 0.19 mm; 95% CI: 0.23-0.45), while the dry canal group showed the highest deviation (0.58 ± 0.22 mm; 95% CI: 0.46-0.70), suggesting comparatively lower accuracy.

**Table 1:** Descriptive statistics of working length deviation (mm) across canal conditions. Values represent mean deviation from actual working length (EWL − AWL) ± standard deviation, CI: Confidence interval, NaOCl: Sodium hypochlorite.

Canal condition	n	Mean (mm)	SD	Minimum	Maximum	95% CI
Normal saline	15	0.21	0.14	0.05	0.49	0.13–0.29
3% NaOCl	15	0.34	0.19	0.09	0.72	0.23–0.45
Dry canal	15	0.58	0.22	0.21	1.02	0.46–0.70

Inter-group comparison using one-way ANOVA (Table 2) revealed a statistically significant difference in the mean deviation among the three canal conditions (F = 18.74, p < 0.001). This indicated that the canal environment significantly influenced the accuracy of the apex locator.

**Table 2:** One-way ANOVA for inter-group comparison of working length deviation. One-way analysis of variance (ANOVA) for inter-group comparison of working length deviation, df: degree of freedom, *p < 0.001 denotes high statistical significance.

Source of variation	Sum of squares (SS)	df	Mean square	F-value	p-value
Between groups	1.248	2	0.624	18.74	< 0.001*
Within groups	1.395	42	0.033		

Post-hoc analysis using Tukey’s HSD test (Table 3) demonstrated statistically significant differences between all pairwise comparisons. The normal saline group showed significantly lower deviation compared to both NaOCl (mean difference = 0.13 mm, p = 0.038) and dry canal (mean difference = 0.37 mm, p = 0.001). Similarly, the NaOCl group exhibited significantly lower deviation than the dry canal group (mean difference = 0.24 mm, p = 0.042).

**Table 3:** Post-hoc Tukey HSD test for pairwise comparisons. Tukey’s Honestly Significant Difference (HSD) test was used for multiple comparisons, *p < 0.05: significant; **p < 0.001: highly significant, NaOCl: Sodium hypochlorite.

Comparison	Mean difference (mm)	t-value	p-value (Tukey)
Normal saline vs. 3% NaOCl	0.13	2.31	0.038*
Normal saline vs. Dry canal	0.37	3.16	0.001**
3% NaOCl vs. Dry canal	0.24	1.91	0.042*

The distribution of the accuracy categories is summarized in Table 4. The proportion of highly accurate measurements (within ±0.5 mm) was greatest in the normal saline group, followed by the NaOCl group and the dry canal group. When considering clinically acceptable measurements (within ±1 mm), the normal saline, NaOCl, and dry canal groups achieved 100%, 93.3%, and 86.7% accuracy, respectively. Although these differences were clinically notable, the chi-square test did not show a statistically significant association between canal condition and accuracy category (χ² = 8.62, p = 0.071).

**Table 4:** Accuracy classification of electronic working length measurements. Accuracy is defined based on deviation from actual working length, Accurate: ±0.5 mm, Acceptable: ±1.0 mm, Inaccurate: > ±1.0 mm, Chi-square test used for comparison, NaOCl: Sodium hypochlorite, p > 0.05 denotes no statistical significance.

Canal condition	n	Accurate (±0.5 mm)	Acceptable (±1.0 mm)	Inaccurate (> ±1.0 mm)	Chi stats (χ²)	p-value
Normal saline	15	14	1	0	8.62	0.071
3% NaOCl	15	11	3	1		
Dry canal	15	7	6	2		

**Figure 1:**
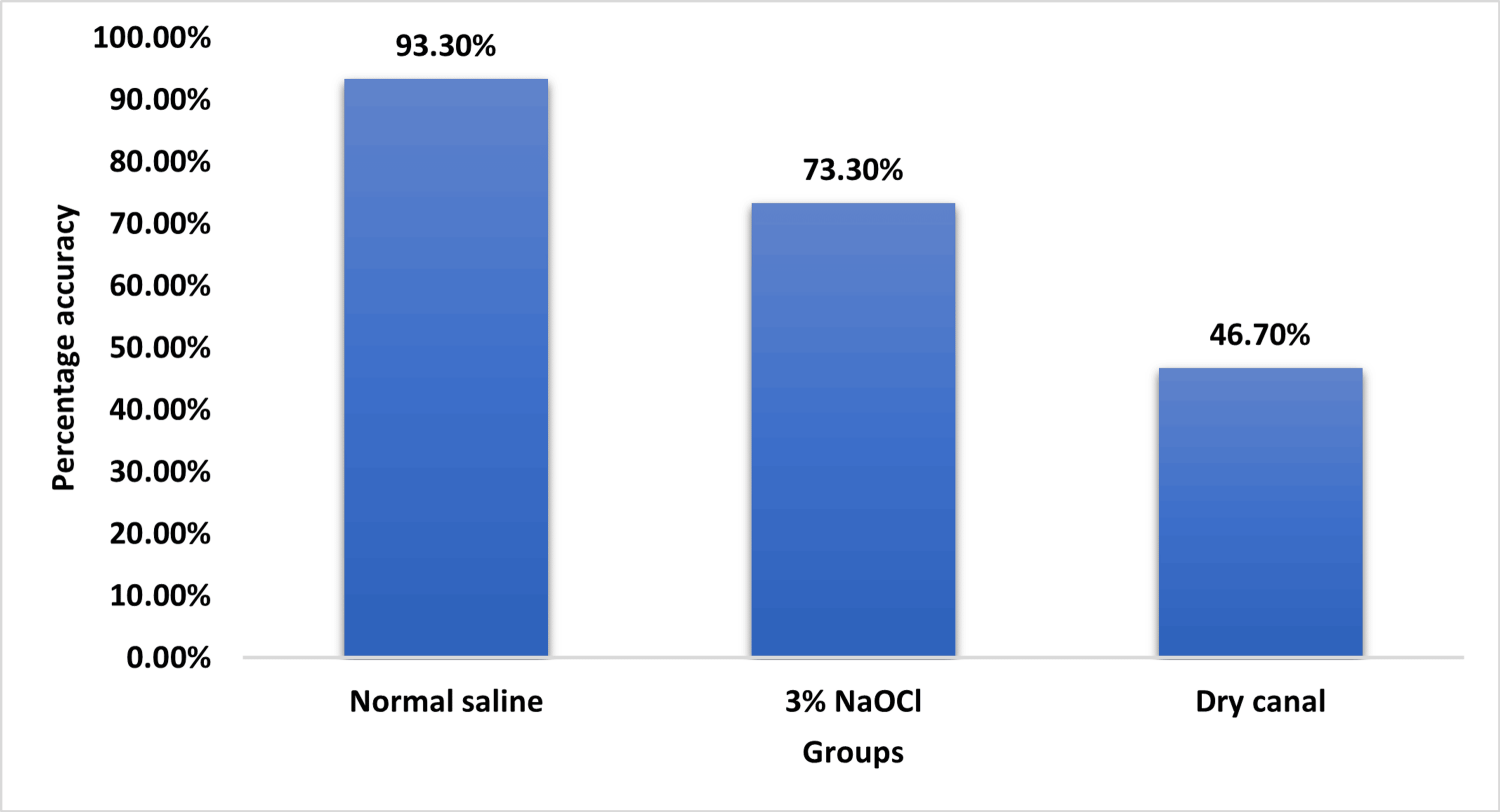
Comparison of mean working length deviation across different canal conditions. NaOCl: Sodium hypochlorite.

Figure 1 illustrates the comparative mean deviation in working length measurements across the three canal conditions. The bar graph clearly demonstrates that the normal saline group had the least deviation, followed by the NaOCl group, whereas the dry canal group exhibited the highest deviation. Statistically significant differences between groups, as determined by post-hoc analysis.

## Discussion

The present in vitro study evaluated the accuracy of a third-generation EAL, Root ZX Mini, under three different canal conditions (dry, normal saline, and 3% NaOCl) by comparing EWL with AWL. The findings demonstrated that the canal environment significantly influenced the accuracy of EAL measurements, with normal saline providing the most accurate results, followed by NaOCl, while the dry canal condition showed the greatest deviation.

The superior accuracy observed for the normal saline group can be attributed to its optimal ionic conductivity. The function of EALs is based on impedance measurements within the root canal system, and the presence of electrolytes facilitates stable electrical conduction [5-7]. Normal saline is a consistent ionic medium that enhances signal transmission, thereby improving the precision of apex detection. This finding is consistent with those of previous studies by Kobayashi and Suda [12], who reported that modern EALs demonstrated improved accuracy in the presence of conductive irrigants. Similarly, Jenkins et al. [10] found that saline conditions yielded more reliable working length measurements than dry canal conditions. Teja et al. [13] demonstrated that both standalone and integrated EAL endomotors provided measurements within clinically acceptable limits, although all readings showed slight negative deviations. The measurement accuracy was significantly influenced by intracanal conditions, with sodium hypochlorite showing improved performance in certain devices, highlighting the effect of irrigants on EAL accuracy.

A systematic review by Shekarbaghani et al. [14] reported that intracanal irrigants significantly influence EAL accuracy, with NaOCl potentially reducing precision owing to its high electrical conductivity. These findings are in agreement with the present study, where NaOCl demonstrated lower accuracy than normal saline, highlighting the impact of irrigant composition on impedance-based measurements. In contrast to the present findings, Bilaiya et al. [5] reported that Root ZX Mini exhibited the highest accuracy in dry canal conditions, with irrigants reducing measurement precision. This discrepancy may be attributed to differences in study design, particularly the use of artificially created root perforations and varying irrigant types, which can alter electrical conductivity patterns and affect impedance-based measurements differently compared with intact root canal systems.

The dry canal condition exhibited the highest mean deviation from AWL. This may be explained by the lack of an adequate electrolytic medium for consistent impedance readings. In a dry environment, the electrical circuit between the file and periodontal simulation becomes unstable, leading to erratic readings and reduced accuracy. These findings align with earlier observations by Gordon and Chandler [15], who emphasized that completely dry canals may compromise the performance of EALs. The findings of the present study are supported by the work of Venturi and Breschi [16], who reported that Root ZX demonstrates greater accuracy in conductive (wet) canal environments, while dry conditions may result in unstable readings. This can be attributed to the device’s reliance on impedance ratio measurements, which require an electrolyte medium for stable signal transmission, thus explaining the higher deviations observed in dry canals in the present study. The variability observed under dry conditions underscores the importance of maintaining a minimally moist canal environment during the clinical measurements.

The NaOCl group demonstrated an intermediate accuracy. Although NaOCl is an electrolyte and supports electrical conduction, its chemical properties may influence the measurement stability. NaOCl is a strong oxidizing agent with variable conductivity depending on its concentration and temperature. This could contribute to fluctuations in impedance and slightly reduced accuracy compared to saline. A previous study by Meares and Steiman [17] similarly reported that NaOCl did not significantly impair EAL function. The findings of the present study are in agreement with those of Khattak et al. [18], who reported that Root ZX maintains high accuracy within clinically acceptable limits in the presence of various irrigants, including normal saline and NaOCl. However, they observed reduced precision with NaOCl compared to saline, which supports the present results and may be attributed to the higher electrical conductivity of NaOCl affecting the impedance stability.

One-way ANOVA revealed a statistically significant difference among the groups, confirming that canal conditions play a crucial role in determining measurement accuracy. Furthermore, Tukey’s post hoc analysis demonstrated that all pairwise comparisons were statistically significant, reinforcing the robustness of these findings. However, despite the clear differences in the mean deviation, the chi-square analysis of categorical accuracy did not reach statistical significance. This discrepancy may be attributed to the relatively small sample size within each group, which limits the statistical power to detect differences in categorical distributions. A scoping review by Golkar et al. [19] highlighted that multiple factors, including intracanal irrigants, canal anatomy, and device-related variables, significantly influence the accuracy of EALs.

Clinical implications

From a clinical perspective, the findings suggest that the presence of a conductive irrigant enhances the reliability of EWL determination. Maintaining a canal environment with normal saline or a similar electrolyte during measurement may improve accuracy and reduce the risk of under- or over-instrumentation. While sodium hypochlorite is routinely used for its antimicrobial properties, clinicians should be aware that its measurement accuracy, although acceptable, may not be optimal compared to that of saline. Importantly, complete drying of the canal before using an EAL should be avoided, as it may compromise measurement precision. These insights support the integration of electronic apex locators as reliable adjuncts to radiographic methods, improving efficiency while minimizing radiation exposure.

Limitations

Despite its strengths, this study had several limitations. First, the in vitro design may not fully replicate the clinical conditions, particularly the complex anatomy and biological variability of periapical tissues. Although alginate was used to simulate the periodontal ligament, it could not perfectly mimic the in vivo electrical characteristics. Second, the sample size, while statistically adequate, may have limited the detection of significant differences in categorical accuracy analysis. Third, only a single EAL model and one concentration of NaOCl were evaluated, which may restrict the generalizability of the findings. In addition, operator-dependent factors, although minimized through standardization and calibration, cannot be eliminated entirely.

## Conclusions

Within the limitations of this in vitro study, the accuracy of electronic working length determination using the Root ZX Mini was significantly influenced by the intracanal conditions. Normal saline provided the most accurate and consistent measurements, followed by 3% sodium hypochlorite, whereas dry canal conditions resulted in the greatest deviation from the actual working length. Although all measurements remained within the clinically acceptable limits, the findings highlight the importance of maintaining a conductive canal environment during apex locator use. Clinically, the use of a moist canal, particularly with saline, may enhance the measurement reliability and improve endodontic treatment outcomes.
